# *Bacillus subtilis* Strain YJ-15, Isolated from the Rhizosphere of Wheat Grown under Saline Conditions, Increases Soil Fertility and Modifies Microbial Community Structure

**DOI:** 10.3390/microorganisms12102023

**Published:** 2024-10-06

**Authors:** Junkang Sui, Chenyu Wang, Pengfei Chu, Changqing Ren, Feifan Hou, Yuxuan Zhang, Xueting Shang, Qiqi Zhao, Xuewen Hua, Hengjia Zhang

**Affiliations:** 1 College of Agriculture and Biology, Liaocheng University, Liaocheng 252000, China; suijunkang@lcu.edu.cn (J.S.); wcy20031214@163.com (C.W.); chupengfei@lcu.edu.cn (P.C.); hff050129@163.com (F.H.); zyx004724@163.com (Y.Z.); sxt031216@163.com (X.S.); tavia123456@163.com (Q.Z.); huaxuewen@lcu.edu.cn (X.H.); 2 Liaocheng Science and Technology Bureau, Liaocheng 252000, China; lcskjjnczx@lc.shandong.cn

**Keywords:** wheat, rhizosphere, salinized land, microbial community, *Bacillus subtilis*

## Abstract

Soil salinization during wheat cultivation considerably diminishes soil fertility and impedes wheat growth, primarily due to rhizosphere microbial community changes. Our study investigates the application of *Bacillus subtilis* YJ-15, a strain isolated from the rhizosphere of wheat cultivated in salinized soil, as a soil remediation agent. This strain has demonstrated significant salt tolerance, disease suppression capabilities, and growth-promoting attributes in previous studies. The wheat rhizosphere was examined to assess the impact of *Bacillus subtilis* YJ-15 on microbial community composition and soil fertility. Fertility of soil in saline soil was significantly increased by inoculating wheat with YJ-15. The microbial community structure within the wheat rhizosphere inoculated with *Bacillus subtilis* YJ-15 was analyzed through sequencing on the Illumina MiSeq platform. Phyla Proteobacteria and Acidobacteria were identified as the dominant bacteria. Basidiomycota, Mortierellomycota, and Ascomycota dominated the fungal phyla. Among the bacterial genera, *Pseudomonas*, *Arthrobacter*, and *Bacillus* were predominant. The predominant fungal genera included *Alternaria*, *Cephalotrichum*, *Mortierella*, and *Chaetomium*. A significant increase in *Gaiella* and *Haliangium* levels was observed in the YJ group compared to the control group. Additionally, the fungal genera *Epicoccum*, *Sporidiobolus*, and Lecythophora have significantly increased in YJ abundance. One of the potential benefits of *Bacillus subtilis* YJ-15 in the cultivation of wheat on salinized land is its ability to enhance the rhizosphere microbial community structure and improve soil fertility.

## 1. Introduction

Soil salinization presents an escalating challenge to agricultural productivity. Inadequate soil management and irrigation practices, in conjunction with the extensive application of chemical fertilizers, have exacerbated the global issue of soil salinization, leading to a continuous reduction in arable land [1]. A large proportion of the world’s soils are salinized, affecting over 800 million hectares, with 20% of the irrigated soils impacted [2]. This problem is particularly pronounced in China, where salinized soil covers 6.62% of the nation’s arable land on average, covering 3.6 × 10^7^ hectares [3]. Soil salinization is increasingly constraining agricultural production globally, thereby exacerbating land degradation [4,5]. A pertinent example is the Yellow River Delta Region (YRD) in China, which is an important food producing region, where 443,000 hectares of land are affected by saline alkalization. This impacted area accounts for nearly half of the region’s arable land, resulting in significant detrimental effects on crop yields [6]. High sodium levels in soil create saline–alkali conditions that are difficult for cultivation [7]. Agricultural productivity is not only adversely affected by salinity and alkali soil, but it is also adversely affected by water storage and soil nutrient availability [8]. Certain ions present in saline soil elements exhibit toxicity and induce alterations in the soil’s physical properties. An elevation in sodium ion concentration diminishes soil porosity, consequently reducing the soil’s capacity to retain essential nutrient elements [9]. A further reduction in functional microbial biomass was observed in farmland soil under salinization and alkalization [10].

Wheat (*Triticum aestivum* L.) is extensively cultivated worldwide, yielding over 800 million tons annually, with China producing around 137 million tons (http://www.fao.org/faostat/, accessed on 1 August 2024). It is estimated that wheat contributes approximately 20% of the daily caloric and protein intake of the global population [11]. However, saline soils have significantly constrained crop yields [12]. Wheat, in particular, is adversely affected by salt stress, which disrupts its physiological processes and inhibits its growth [13]. There is a reduction in leaf and root growth in soil solution when salt is present. The reduction in stomatal conductance also inhibits photosynthesis. Agricultural productivity is diminished due to soil salinization’s adverse effects [14]. Despite these negative impacts, saline–alkali environments significantly inhibit the growth of microorganisms [15]. As the most active components in soil, microorganisms and enzymes play an important role in determining soil organic carbon dynamics. Furthermore, soil salinization exerts a notably detrimental effect on the functionality of these microorganisms [16].

By selecting or developing salt-tolerant crop varieties, we can address some of the challenges to improving crop quality and yield in saline–alkaline soils [17]. However, this approach does not ameliorate soil fertility. Implementing biological improvement methods is necessary to improve saline–alkali land’s utilization. Planting certain improved plant varieties on saline–alkali land can further augment land use efficiency [18]. Several widely cultivated economic crops, such as cotton, exhibit strong adaptability to saline–alkali soils and are thus employed as pioneer crops for ameliorating such lands [19]. Additionally, *Thinopyrum intermedium*, a perennial cross-pollinated species belonging to the wheat family, is extensively cultivated as forage globally. This species is also considered ideal for soil and water conservation as well as for the improvement of saline–alkali soils [20]. While these approaches represent positive efforts, they have not fundamentally resolved the issue of soil salinization. It is important to recognize that soil salinization is a process and is not irreversible. To mitigate soil salinization and optimize the development and utilization of salinized land, it is imperative to conduct comprehensive studies on the properties of salinized soils and systematically monitor their dynamic changes [21]. Conversely, numerous studies aimed at improving salinized land have predominantly focused on the soil in which crops are cultivated. Research has demonstrated that the application of organic fertilizers can ameliorate severely saline–alkali land and enhance rice growth by increasing soil bacterial diversity [22]. It has been demonstrated that biochar and organic fertilizer can improve corn yields in saline–alkali soils of the Yellow River Delta [23]. Concurrently, the use of organic fertilizer enhances soil phosphorus (P) availability and retention capacity, with lower amounts of organic fertilizer proving effective in improving these properties in saline–alkaline soils [22]. Furthermore, the microbial components present in organic fertilizers are critically important for ameliorating soil salinization. The combined use of effective microorganisms and biochar has been demonstrated to mitigate soil salinity, boost soil fertility, and support plant growth by enhancing nutrient absorption and enzyme activities [4]. Desalination of soil could be promoted by *Bacillus subtilis* by increasing soil water retention capacity [24].

However, the underlying mechanisms driving changes in soil microbial communities remain inadequately understood. In our study, we addressed soil salinization by employing a highly efficient, salt-tolerant strain of Bacillus subtilis, and investigated its effects on soil microbial community structure, with a particular focus on its role in soil fertility enhancement.

## 2. Materials and Methods

### 2.1. Research Location and Experimental Setup

Situated in Shandong Province in eastern China, the study site is specifically located in Liaocheng. This region experiences a warm temperate monsoon climate and exhibits characteristics of a semi-arid continental climate. With an average of 2463.0 to 2741.8 h of sunshine per year, this region enjoys favorable climatic conditions. Liaocheng’s annual average temperature is 12.8–13.4 °C, and the annual precipitation is 567.7–637.3 mm. The average relative humidity ranges from 56% to 68%. Furthermore, the frost-free period extends for approximately 200 days, predominantly influenced by southerly and south-southeasterly winds.

The experimental setup was established on the experimental field from Liaocheng Chuangju Fengwanjiang Agricultural Technology Development Co., Ltd. (115.77° E, 36.53° N) in Dongchangfu district, Liaocheng, China in October 2023. Approximately 20 × 60 m rectangular treatment areas were set up along a south-to-north axis. The experimental design employed a block layout. During mechanical cultivation, ridges were eliminated from each plot. A 20 m distance separated the YJ treatment group from the control group (CK). A ‘Jimai 22’ cultivar of winter wheat (*Triticum aestivum* L.) was planted in October 2022 and harvested in June 2023. The soil was treated with a *Bacillus subtilis* YJ-15 suspension at a concentration of 6.37 × 10^8^ CFU/mL. This treatment was applied 15 days before planting and subsequently administered twice post-planting, specifically on March 6 and May 6, at a rate of 200 kg/ha applied over the entire area each time. The fermentation medium for *Bacillus subtilis* YJ-15 consisted of 45 g/L corn flour, 25 g/L corn syrup, 15 g/L glucose, 8 g/L NaCl, 1.5 g/L MnSO_4_, and distilled water. Effective water and fertilizer management practices are crucial throughout the wheat growth period.

### 2.2. Soil Sampling and Fertility Assessment

The root samples were collected on 27 May 2023, at a depth of 15–20 cm. During soil removal, bulk soil was cleared, and rhizosphere soil was identified as the soil attached to the roots. Five replicates for each group were obtained using simple random sampling. For subsequent high-throughput sequencing and soil fertility analysis, rhizosphere soil samples from wheat treated with Bacillus subtilis YJ-15 (YJ) and the control group (CK) were collected. Kjeldahl nitrogen determination and sulfuric acid digestion were used to determine the total nitrogen content of soil (TN). The content of total phosphorus (TP) was assessed using a molybdenum–antimony spectrophotometer at the alkali melting temperature in NaOH. A NaOH alkali fusion flame photometer was used to determine the total potassium content (TK). In conjunction with the molybdenum–antimony colorimetric method, sodium bicarbonate/sodium fluoride hydrochloric acid extraction was used to determine the availability of phosphorus (AP). To determine the readily available potassium content (AK), ammonium acetate extraction was conducted and flame photometry was applied. Soil nitrate nitrogen (NN) and ammonium nitrogen (AN) concentrations in the soil were quantified using potassium chloride solution extraction, with subsequent analysis by dual-wavelength colorimetry for NN and indophenol blue colorimetry for AN. A volumetric measurement of organic matter (organic carbon) (OC) was conducted with potassium dichromate, using external heating. By using chloroform fumigation extraction, soil microbial biomass carbon (MBC) was quantified. The pH levels were assessed with a pH meter, while the electrical conductivity (EC) values were determined using an EC meter.

### 2.3. DNA Extraction and PCR

The E.Z.N.A.^®^ Soil DNA Kit (Omega Bio-tek, Norcross, GA, USA) was used to extract total microbial genomic DNA from soil samples. Following 1.0% agarose gel electrophoresis, the quality and concentration of the extracted DNA was determined using a NanoDrop^®^ ND-2000 spectrophotometer (Thermo Scientific Inc., Waltham, MA, USA). For further analysis, DNA samples were stored at −80 °C. This fragment of the bacterial 16S rRNA gene is hypervariable in the V3-V4 region, and the primers 338F (5′-ACTCCTACGGGAGGCAGCAG-3′) and 806R (5′-GGACTACHVGGGTWTCTAAT-3′) [25] have been used to amplify it using an ABI GeneAmp^®^ 9700 PCR thermocycler. Primer pairs ITS1F (5′-CTTGGTCATTTAGAGGAAGTAA-3′) and ITS2R (5′-GCTGCGTTCTTCATCGATGC-3′) were used to amplify the hypervariable region of the ITS rRNA gene. The PCR reaction mixture comprised 2 μL of 2.5 mM dNTPs, 4 μL of Fast Pfu polymerase, 4 μL of 5× Fast Pfu buffer, 0.8 μL of 5 μM each primer, 0.4 μL of Fast Pfu polymerase, 10 ng of template DNA, and ddH_2_O to a total volume of 20 μL. Amplification conditions were as follows: an initial denaturation at 95 °C for 3 min, followed by 27 cycles of denaturation at 95 °C for 30 s, annealing at 55 °C for 30 s, and extension at 72 °C for 45 s. The reaction concluded with a final extension at 72 °C for 10 min, followed by cooling to 4 °C. Triplicate amplifications were performed on all the samples. An AxyPrep DNA Gel Extraction Kit (Axygen Biosciences, Union City, CA, USA) was used to remove the PCR products from a 2% agarose gel and purify them, and then Quantus^TM^ Fluorometers (Promega, Madison, WI, USA) were used to quantify them.

### 2.4. Illumina MiSeq Sequencing

Paired-end amplicons, pooled in equimolar quantities, were sequenced using the Illumina MiSeq PE300 platform (Illumina, San Diego, CA, USA) according to Majorbio Bio-Pharm’s standard protocols. The raw reads are available in the NCBI database under BioProject PRJNA1138005.

### 2.5. Data Processing

Raw FASTQ files were processed using a custom Perl script for demultiplexing, filtered with fastp 0.19.6 [26] and merged using FLASH 1.2.7 [27]. The processing criteria were as follows: (i) whenever a quality score was below 20, a 300-bp read was truncated; all reads shorter than 50 bp, as well as any with opaque characters, were discarded from the analysis; (ii) in order to assemble sequences over 10 bp, at least 0.2 mismatches were required in their overlap region. Less than 0.2 mismatches were rejected; (iii) barcodes and primers were used to differentiate samples, using sequence direction adjustments to match barcodes and primers with up to two nucleotide mismatches. Following the optimization of the sequences, UPARSE 7.1 was used to cluster them into operational taxonomic units (OTUs) based on sequence similarity scores of 97% [28,29]. Representative sequences were chosen for each operational taxonomic unit (OTU). In each sample, the 16S rRNA gene sequences were rarefied to mitigate the impact of the sequencing depth on alpha and beta diversity metrics. Good’s coverage averaged 99% using this approach.

### 2.6. Statistical Analysis

The Majorbio Cloud platform (https://cloud.majorbio.com, accessed on 23 May 2024) was used to analyze the soil microbiota via bioinformatic analysis. With Mothur v1.30.1, rarefaction curves and alpha diversity indices were computed, including the OTUs observed, the Shannon index, Chao1 richness, and Good’s coverage [30]. Using Vegan v2.5-3, a principal coordinate analysis (PCoA) based on the Bray–Curtis dissimilarity was used to determine the similarity between microbial communities across samples. The PERMANOVA test in the Vegan v2.5-3 package quantified the treatment’s contribution to variation. Furthermore, we determined significant abundances of bacteria by phylum and genus composition by using linear discriminant analysis effect size (LEfSe) [31].

In the results, the means are presented along with the standard deviations (SD). We calculated the significance levels for the soil fertility, diversity, and richness indices between YJ and CK groups at significance levels of *p* < 0.05 or *p* < 0.01 with a one-way ANOVA. SAS, version 9 (SAS Institute Inc., Cary, NC, USA), was used to conduct all the statistical analyses.

## 3. Results

### 3.1. Soil Fertility Variation

The soil fertility factor analysis indicated significant increases (*p* < 0.01) in total nitrogen (TN), total potassium (TK), available phosphorus (AP), available potassium (AK), and available nitrate nitrogen (NN) in the *Bacillus subtilis* YJ-15-treated group (YJ) compared to the control group (CK). A significant increase in microbial biomass carbon (MBC) and total phosphorus (TP) was also observed in the YJ group (*p* < 0.05). On the other hand, no significant differences in ammonium nitrogen (AN) and organic carbon (OC) were observed between the groups. (Table 1).

### 3.2. Sequencing Quality Evaluation

**Figure 1:**
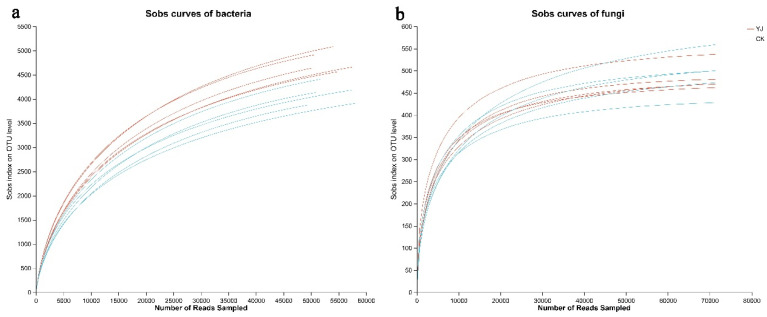
The bacterial (**a**) and fungal (**b**) species sobs curves were analyzed to evaluate the effect of a 3% dissimilarity threshold on the identification of unobserved OTUs. “YJ” represents the treated group with the bacterial agent, whereas “CK” represents the control group without the bacterial agent applied.

**Figure 2:**
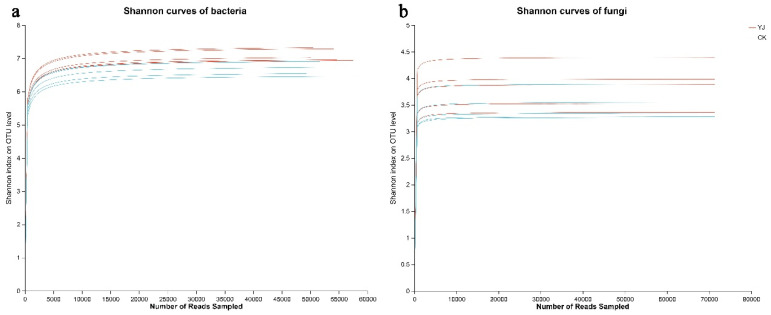
Shannon curves for bacteria (**a**) and fungi (**b**). “YJ” represents the treated group with the bacterial agent, whereas “CK” represents the control group without the bacterial agent applied.

In accordance with the sequencing results, the YJ and CK groups obtained 77,959 and 76,990 bacterial 16S rDNA sequences, respectively, as well as 77,961 and 93,428 fungal ITS sequences. We classified the reads into distinct OTUs based on their clustering dissimilarity threshold of 3%. At a distance of 0.03, neither the bacterial nor fungal diversity rarefaction curves reached a plateau under Sobs (Figure 1), suggesting that they do not reflect the full diversity of the community in the sequencing data. When the Shannon diversity index is combined with rarefaction curves, a more comprehensive analysis of community diversity can be completed (Figure 2). Increasing the number of reads resulted in plateauing Shannon diversity curves, indicating that enough data had been collected to analyze community diversity.

### 3.3. α-Diversity and β-Diversity Analysis

In the soil samples, the indicators of diversity and richness (Table 2) indicated that the bacterial communities of the YJ and CK groups were comparable in terms of richness and diversity. As compared to the CK group, the ACE, Chao, and Sobs values increased significantly (*p* < 0.01) in the YJ rhizosphere bacterial community. YJ and CK had significantly different Shannon diversity indices (*p* > 0.05). Although there was no significant difference in Simpson diversity indices between YJ and CK, YJ’s diversity index was lower. Overall, a more diverse bacterial community was found in the YJ group based on both the Shannon and Simpson indices (Table 2).

Fungal community diversity and richness values for the YJ group were lower than those for the CK group using ACE, Chao, and Sobs; however, these differences were not statistically significant. Similarly, a higher Shannon index indicates a greater degree of diversity, while a lower Simpson index indicates a lesser degree; slightly higher fungal diversity was indicated in the YJ group, but these differences were also not statistically significant.

**Figure 3:**
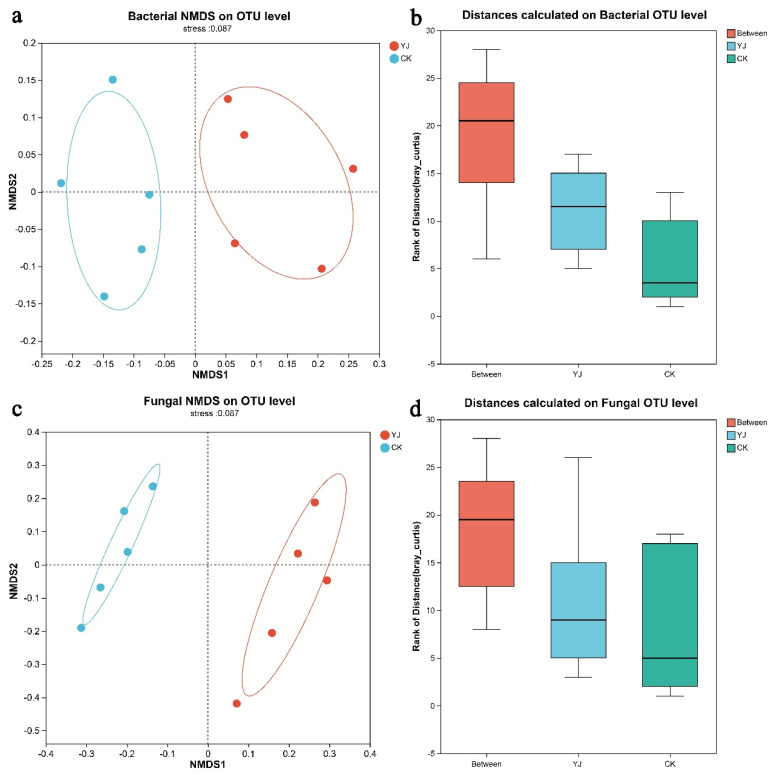
NMDS analysis and ANOSIM analysis of rhizosphere soil microbes from non-deep tillage and deep tillage wheat cultivation. (**a**) Bacterial NMDS analysis on OTU level, (**b**) Bacterial ANOSIM analysis on OTU level, (**c**) Fungal NMDS analysis on OTU level, (**d**) Fungal ANOSIM analysis on OTU level. “YJ” represents the treated group with the bacterial agent, whereas “CK” represents the control group without the bacterial agent applied.

A comparative analysis of species diversity across various microbial communities was conducted to examine the similarities and differences between samples from different groups. The Non-Metric Multidimensional Scaling (NMDS) analysis yielded a stress value indicative of an ordination with a clear definition and representation. A significant level of dispersion and aggregation of the communities was observed between the YJ and CK groups, suggesting significant aggregation and dispersion within the communities (Figure 3a,c). Additionally, an analysis of similarities (ANOSIM) confirmed that there was a significantly higher dissimilarity between the two groups than within them (Figure 3b,d).

### 3.4. Composition and Structure of Microbial Communities

**Figure 4:**
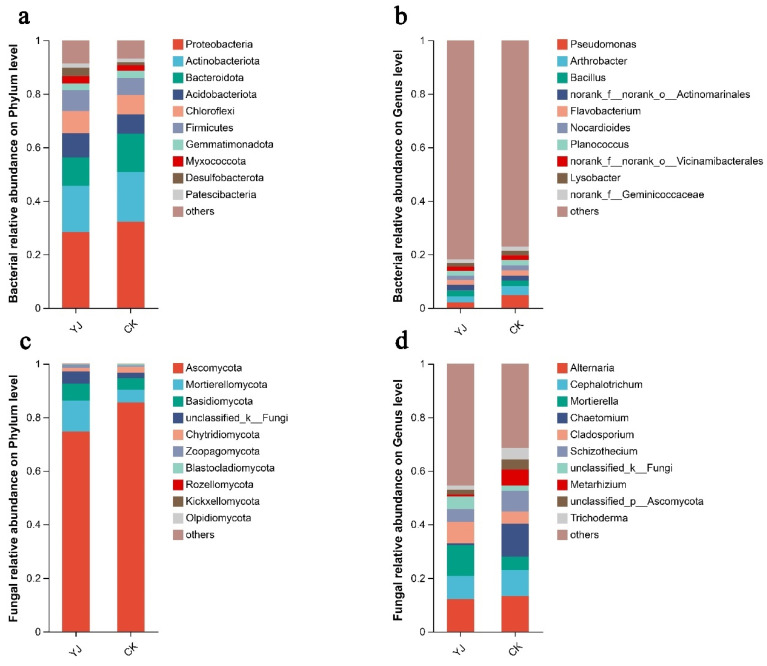
Bacterial and fungal communities’ composition. (**a**) Phylum level of bacterial composition. (**b**) Genus level of bacterial composition. (**c**) Phylum level of fungal composition. (**d**) Genus level of fungal composition. Major genera are represented by stacked bar graphs. “YJ” represents the treated group with the bacterial agent, whereas “CK” represents the control group without the bacterial agent applied.

The sequences were taxonomically classified using Mothur software v1.30.1. According to the phylum level, Proteobacteria, Actinobacteria, Bacteriaroidota, Acidobacteriota, and Chloroflexi are the major bacterial taxa identified in rhizosphere soil, which collectively accounted for over 81% of the total phyla abundance of bacteria (Figure 4a). Specifically, the Proteobacteria phylum constituted 28.38% and 32.26% in the YJ and CK groups, respectively. Actinobacteria accounted for 17.28% and 18.61%, respectively, while Bacteroidota accounted for 10.53% and 14.30%, respectively. There were 9.12% of Acidobacteriota in YJ and 7.15% in CK. Ascomycota, Basidiomycota, Mortierellomycota, and Chytridiomycota accounted for over 93% of the total abundance of fungal phyla (Figure 4c). Specifically, the Ascomycota constituted 74.67% and 85.53% of the fungal communities, representing the largest phylum in the YJ and CK groups, respectively. YJ showed a relative abundance of 11.50% for Mortierellomycota, and CK had a relative abundance of 4.87%. Basidiomycota exhibited relative abundances of 6.39% and 4.27% in the YJ and CK groups, respectively.

Both the groups had similar bacterial compositions at the genus level, although individual genera exhibited differential distributions. Excluding unidentified genera, *Pseudomonas*, *Arthrobacter*, and *Bacillus* were the predominant genera (Figure 4b). The *Pseudomonas* genus accounted for 2.05% of the bacterial communities in the YJ group and 4.75% in the CK group. *Arthrobacter* showed relative abundances of 2.19% and 3.45%, respectively, while *Bacillus* accounted for 2.25% and 2.09%, respectively. Among genera, there were greater differences in fungal composition. The genera *Alternaria*, *Cephalotrichum*, *Mortierella*, and *Chaetomium* were predominant (Figure 4d). Specifically, the genus *Alternaria* constituted 12.14% and 13.27% of the fungal communities in the YJ and CK groups, respectively. *Cephalotrichum* showed relative abundance of 8.70% and 9.84%, respectively. *Mortierella* indicated 11.47% and 4.84% relative abundance, respectively. Lastly, *Chaetomium* represented 0.61% and 12.22% of the fungal community, respectively.

**Figure 5:**
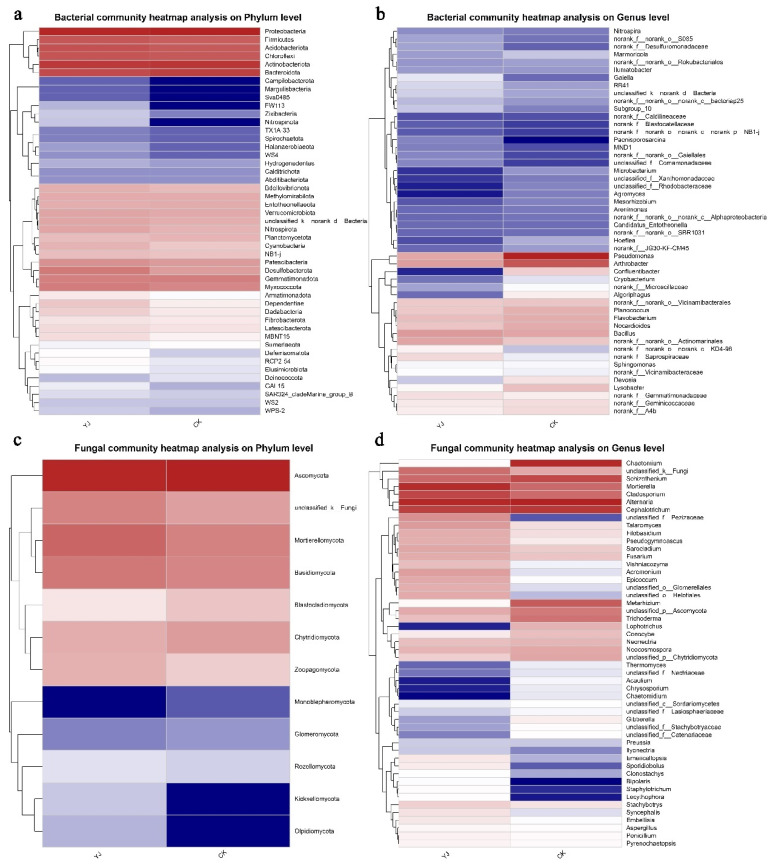
Distributions of bacteria and fungi grouped hierarchically. (**a**) Phylum level of bacterial taxonomic composition. (**b**) Genus level of bacterial taxonomic composition. (**c**) Phylum level of fungal taxonomic composition. (**d**) Genus level of fungal taxonomic composition. “YJ” represents the treated group with the bacterial agent, whereas “CK” represents the control group without the bacterial agent applied.

According to the heatmap, the hierarchical clustering of bacterial and fungal distributions confirms the community bar plot findings. Specifically, they confirm that Proteobacteria, Actinobacteria, Bacteroidota, Firmicutes, Acidobacteria, and Chloroflexi are the predominant phyla within both groups. Additionally, the CK group exhibits minimal presence of Campilobacterota and Margulisbacteria, while the YJ group shows a negligible amount of Deinococcota (Figure 5a). The genera *Pseudomonas*, *Arthrobacter*, *Nocardioides*, and *Bacillus* exhibit the highest relative abundance among bacterial genera in both groups. Conversely, the genera *Confluentibacter* and *Devosia* demonstrate notably low content in the YJ group, while the *Paenisporosarcina* shows particularly low content in the CK group (Figure 5b).

The Ascomycota and Mortierellomycota phyla exhibit the highest relative abundance. Conversely, the Kickxellomycota and Olpidiomycota phyla demonstrated notably low relative abundance within the CK group, while the Monoblepharomycota phylum displayed similarly low content in the YJ group (Figure 5c). The *Alternaria*, *Cephalotrichum*, *Schizothecium*, *Chaetomium* and *Mortierella* in bacterial genera exhibit higher relative abundances at the genus level compared to others in both groups. Notably, *Chaetomium* content is significantly higher in the CK group compared to the YJ group. *Bipolaris*, *Staphylotrichum*, and *Lecythophora*, on the other hand, exhibit low content in the CK group, while *Lophotrichus*, *Acaulium*, *Chrysosporium*, and *Chaetomidium* are particular low in the YJ group (Figure 5d).

**Figure 6:**
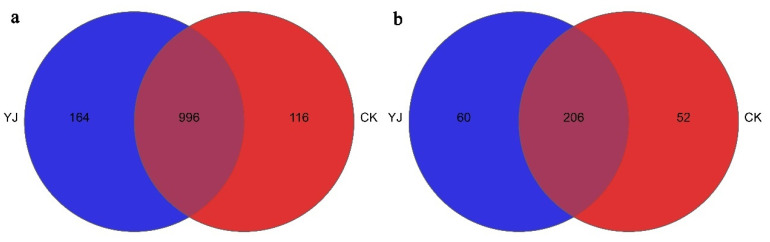
Venn diagram with unique and shared genera for (**a**) bacteria and (**b**) fungi. “YJ” represents the treated group with the bacterial agent, whereas “CK” represents the control group without the bacterial agent applied.

A total of 1160 bacterial genera were observed in the YJ treatment group, whereas 1112 fungal genera were observed in the CK treatment group, as shown in Figure 6. It was noted that three predominant genera—*Pseudomonas*, *Arthrobacter*, and *Bacillus*—were found in both YJ and CK groups. In contrast, the genera *Syntrophus*, *Sedimenticola*, and *Dehalogenimonas* were unique to the YJ group, while *Aetherobacter*, *Tundrisphaera*, and *Owenweeksia* were exclusively observed in the CK group.

A comprehensive analysis revealed the identification of 266 and 258 genera in the YJ and CK groups, respectively. Of these, 206 genera were common to both groups, with *Alternaria*, *Cephalotrichum*, and *Mortierella* being the predominant shared genera. In contrast, the genera *Didymosphaeria*, *Trematosphaeria*, *Phaeomyces*, and *Limonomyces* were uniquely present in the YJ group, whereas *Scopulariopsis*, *Veronaea*, *Geomyces*, and *Ascobolus* were exclusively found in the CK group.

**Figure 7:**
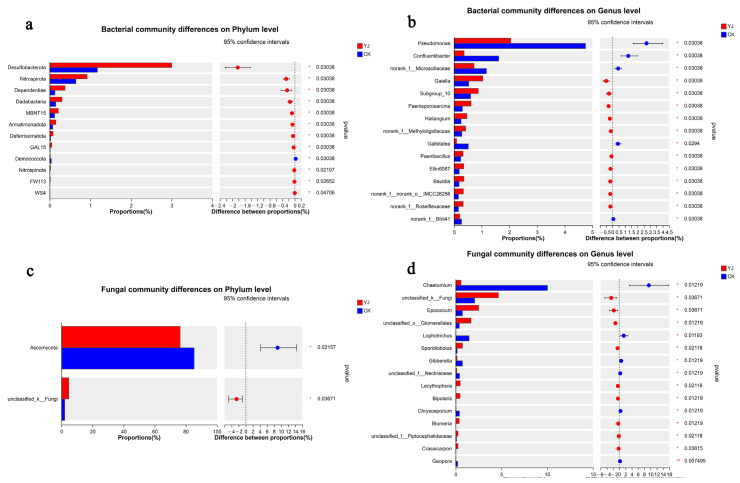
Significant test of differences between two groups. (**a**) Phylum-level bacterial significant differences (**b**) Genus-level bacterial significant differences (**c**) Phylum-level fungal significant differences (**d**) Genus-level fungal significant differences. “YJ” represents the treated group with the bacterial agent, whereas “CK” represents the control group without applied the bacterial agent.

Various microorganisms may respond to environmental changes as key species. The YJ and CK groups showed significant differences in the relative abundance of Desulfobacterota, Nitrospirota, Dependentiae, and Dadabacteria at the bacterial phylum level (*p* < 0.05). A significant difference was observed between the CK and YJ groups when it came to the content of Deinococcota (*p* < 0.05) (Figure 7a). The relative abundance in the bacterial genera of *Gaiella*, *Paenisporosarcina*, *Haliangium*, and *Paenibacillus* significantly increased in the YJ group (*p* < 0.05). Conversely, the CK group exhibited significantly higher content of *Pseudomonas* and *Confluentibacter*. (*p* < 0.05) (Figure 7b).

There were significant differences between the CK and YJ groups in the proportion of Ascomycota at the phylum level (*p* < 0.05). (Figure 7c). The relative abundance of fungal genera, *Epicoccum*, *Sporidiobolus*, and *Lecythophora* were significantly elevated in the YJ group (*p* < 0.05). Conversely, YJ showed significant reductions in the relative abundances of *Chaetomium*, *Lophotrichus*, *Gibberella*, and *Chrysosporium* (*p* < 0.05) (Figure 7d).

**Figure 8:**
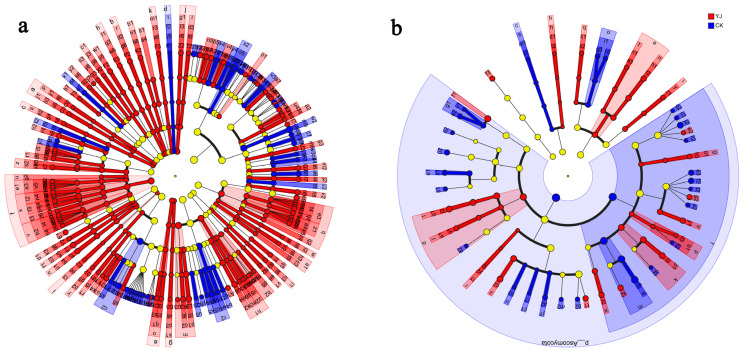
Multilevel species differences evaluated through LEfSe analysis. (**a**) Bacterial multi-level species differences. (**b**) Fungal multi-level species differences. Nodes of different colors signify microbial communities that are significantly enriched in their respective groups and contribute notably to inter-group differences.

Based on the LEfSe analysis, differential tests were conducted on wheat grown under deep tillage cultivation versus non-deep tillage cultivation. The results indicated that the bacterial genera *Gaiella*, *Paenisporosarcina*, *Haliangium*, *Bryobacter*, *Aquicella*, and *Bauldia* were specific to the YJ group, whereas the genera *Pseudomonas*, *Confluentibacter*, *Galbitalea*, *Nitrosospira*, *Agromyces*, and *Hoeflea* were specific to the CK group (Figure 8a). In the YJ group, the fungal genera *Epicoccum*, *Lecythophora*, *Sporidiobolus*, *Staphylotrichum*, *Bipolaris*, and *Clonostachys* were identified as specific, whereas in the CK group, the genera *Chaetomium*, *Lophotrichus*, *Boubovia*, *Scopulariopsis*, *Microdochium*, and *Kernia* were found to be specific (Figure 8b).

## 4. Discussion

Rhizosphere microorganisms have been demonstrated to enhance plant stress resistance, with specific beneficial strains significantly promoting crop growth under salt stress conditions [32]. However, in soils with high salinity, microbial activity is inhibited [33]. Farmland ecosystems are heavily dependent on microbial activity to maintain soil fertility. Microorganisms play crucial roles in soil improvement, and the structure of the microbial community is pivotal for soil fertility and plant growth [34,35]. *Bacillus subtilis* is a widely occurring PGPR in nature that has gradually been used in agriculture because of its ability to enhance the rhizosphere of crops, to promote nutrient absorption, and to increase output [36]. Additionally, Bacillus subtilis can improve soil aggregate structure, increase soil water retention capacity, and reduce soil salinity, and then increase wheat yield [37]. In addition to promoting soil desalination, *Bacillus subtilis* may increase soil water retention capability [38]. The soil at the test site is characterized as slightly acidic saline soil. An analysis of the soil fertility indicates that the microbial agents significantly increase the levels of total phosphorus, total nitrogen, total potassium, available potassium, available phosphorus, ammonium nitrogen, and microbial biomass carbon. Compared to previous reports on *Bacillus subtilis*, this strain shows a superior ability to improve soil fertility [39]. Additionally, it reduces the soil’s electrical conductivity (EC) value, thereby demonstrating a positive impact of *B. subtilis* YJ-15. Experiments conducted in the field can better test the positive role of this strain in improving soil salinization compared to laboratory conditions [40]. These findings align with prior research on the amelioration of saline–alkali soils in the Hetao irrigation area of Inner Mongolia. Previous studies show that microbial inoculants can reduce soil salinity and pH, while increasing available potassium, alkaline nitrogen, and effective phosphorus in rhizosphere soil [41].

The sequencing data provided robust support for the analysis of microbial community structure. The data indicated that bacterial species richness and diversity increased significantly in wheat rhizosphere soil treated with *B. subtilis* YJ-15 compared to the control group. Conversely, fungal richness and diversity did not exhibit significant differences between the YJ-15 treated group and the control group, with only a slight reduction noted in the YJ-15 group. Substantial evidence exists suggesting that inoculation with beneficial microorganisms can enhance soil microbial community structure [42,43]. Simultaneously, research has demonstrated that the utilization of various microbial agents positively affects the functionality of soil and its microbial communities. By adding exogenous microbial inoculants, composting processes can be extended at elevated temperatures, enhancing bacterial and fungal diversity and richness [44].

Furthermore, the OTU level microbial community distribution analysis by β-diversity metrics revealed significant intergroup differences surpassing intragroup variations. The application of *B. subtilis* YJ-15 had a substantial impact on the structure of soil microbial communities, according to Nonmetric Multidimensional Scaling (NMDS) analysis. Microbiological communities are commonly studied using this method to determine similarities and differences [45,46]. In addition, ANOSIM analysis revealed that the rhizosphere soil microbial communities in the YJ and CK groups differed significantly more than the dissimilarities observed within each group. It is generally used to identify differences between groups as well as within them through ANOSIM analysis [47,48].

As a result of examining the microbial community structure, both the YJ and CK groups developed a predominance of Proteobacteria, Actinobacteria, Bacteroidota, and Acidobacteriota. These phyla are acknowledged as antagonistic microorganisms that are prevalent in most soil environments [49]. Specifically within soil rhizospheres and root zones, Proteobacteria were dominant bacterial phyla. [50]. Furthermore, the soil ecosystem is particularly rich in Acidobacteria [51]. Additionally, research shows they contribute to soil nitrogen availability and the degradation of plants and microbes’ polysaccharides [52]. There was a significant increase in Desulfobacterota and Nitrospirota abundance in the YJ group, which may be related to sulfur cycling [53].

Sequencing analysis showed that *Pseudomonas*, *Arthrobacter*, and *Bacillus* were the dominant genera in both the YJ and CK rhizospheres, consistent with their known prevalence in similar settings. Notably, *Pseudomonas* and *Bacillus* genera benefit soil and plants by producing enzymes that control plant pathogens [54,55,56]. The notably higher abundance of *Gaiella* and *Haliangium* in the YJ group may positively influence soil fertility, specifically regarding total phosphorus (TP), total nitrogen (TN) and total potassium (TK) contents [57]. According to our analysis of soil fertility, this observation is consistent.

This study found that the most prevalent fungal phyla in soil are Ascomycota, Mortierellomycota, Basidiomycota, and Chytridiomycota, confirming earlier research [50,58,59]. Notably, Ascomycota’s relative abundance increased significantly in the YJ group. There has been some evidence that Ascomycota members can be used as biocontrol agents [60]. The primary fungal genus Mortierella is vital for bioremediation [61], and capable of degrading organic pollutants and oxidizing carbon monoxide [62]. In the YJ group, *Lecythophora* relative abundance increased significantly. Previous studies have reported that *Lecythophora* species possess the ability to degrade polycyclic aromatic hydrocarbons [63].

## 5. Conclusions

Through the application of *Bacillus subtilis* YJ-15 fermentation broth, we observed a significant improvement in soil fertility within salinized areas and an enhancement of the microbial community structure in the rhizosphere. Additionally, our findings suggest that *Bacillus subtilis* YJ-15 holds potential as an environmentally compatible agent for soil remediation. This discovery presents significant potential for the advancement of specialized microbial fertilizers designed to enhance soil quality in saline–alkali soils. Implementing this approach could provide a cost-effective and environmentally sustainable method for augmenting soil productivity in saline–alkali agricultural areas, leveraging the benefits of agricultural microbial technology. Removing soil salinization is a relatively lengthy process [64,65]. Due to the fact that *Bacillus subtilis* YJ-15 has shown a certain ability to relieve soil salinization, the continued use of *Bacillus subtilis* YJ-15 in subsequent planting seasons is expected to play an important role in soil remediation. In future planting seasons, we will continue to study the interaction between *Bacillus subtilis* YJ-15 and soil components, including enzyme activity, nutrient cycling, or microbial interactions in the rhizosphere. In this way, we could track the long-term effects of *Bacillus subtilis* YJ-15 application on soil, to provide more compelling evidence of its efficacy.

## Figures and Tables

**Table 1:** Soil fertility characteristics of deep tillage cultivation and control group.

	TN g/kg	TP g/kg	TKg/kg	AP mg/kg	AK mg/kg	NN mg/kg	AN mg/kg	OC g/kg	MBC mg/kg	pH	EC
YJ	0.82 ± 0.08 A	0.68 ± 0.10 a	19.29 ± 0.39 A	8.95 ± 1.91 A	114.59 ± 4.99 A	121.68 ± 25.04 A	11.69 ± 1.83 a	13.86 ± 2.28 a	432.58 ± 106.84 a	6.20 ± 0.12 A	1.34 ± 0.11 B
CK	0.62 ± 0.09 B	0.54 ± 0.06 b	18.03 ± 0.53 B	5.21 ± 1.37 B	78.63 ± 7.56 B	94.33 ± 2.32 B	11.21 ± 0.93 a	10.18 ± 2.90 a	328.59 ± 118.64 b	5.78 ± 0.08 B	2.64 ± 0.32 A

The data are presented as mean ± standard error (SE), with statistical significance indicated by lowercase letters for *p* < 0.05 and uppercase letters for *p* < 0.01 within the same column. “YJ” represents the treated group with the bacterial agent, whereas “CK” represents the control group without the bacterial agent applied.

**Table 2:** The indices of diversity and richness pertaining to bacterial and fungal communities.

	Sample	ACE	Chao	Sobs	Simpson	Shannon	Coverage
Bacterial	YJ	5672.20 ± 262.04 A	5467.85 ± 228.87 A	4646.40 ± 233.96 A	0.0033 ± 0.0016 a	7.10 ± 0.18 a	0.9744
	CK	4944.62 ± 230.38 B	4757.75 ± 207.22 B	4040.40 ± 214.15 B	0.0065 ± 0.0034 a	6.70 ± 0.21 b	0.9772
Fungal	YJ	494.92 ± 31.96 a	493.95 ± 33.03 a	484.00 ± 30.39 a	0.073 ± 0.045 a	3.83 ± 0.41 a	0.9996
	CK	516.60 ± 60.92 a	516.85 ± 61.68 a	492.00 ± 47.63 a	0.093 ± 0.030 a	3.46 ± 0.26 a	0.9993

The data are presented as mean ± standard error (SE), with statistical significance indicated by lowercase letters for *p* < 0.05 and uppercase letters for *p* < 0.01 within the same column. “YJ” represents the treated group with the bacterial agent, whereas “CK” represents the control group without the bacterial agent applied.

## Data Availability

The raw sequencing data was submitted to the NCBI database BioProject accession number PRJNA1138005.
